# Paramedic literature search filters: optimised for clinicians and academics

**DOI:** 10.1186/s12911-017-0544-z

**Published:** 2017-10-11

**Authors:** Alexander Olaussen, William Semple, Alaa Oteir, Paula Todd, Brett Williams

**Affiliations:** 1 Department of Community Emergency Health and Paramedic Practice, Monash University, Melbourne, Australia; 2 Emergency & Trauma Centre, The Alfred Hospital, Melbourne, Australia; 3 Trauma Service, The Alfred Hospital, Melbourne, Australia; 4 National Trauma Research Institute, The Alfred Hospital, Melbourne, Australia; 5 Paramedic Program, Department of Allied Medical Sciences, Jordan University of Science and Technology, Irbid, Jordan; 6 Department of Community Emergency Health & Paramedic Practice, Monash University, Peninsula Campus, PO Box 527, McMahons Road, Frankston, VIC 3199 Australia; 7 Division of Paramedicine, School of Medicine, University of Tasmania, Hobart, Australia; 8 Monash University Library, Melbourne, Australia

**Keywords:** Search filter, Paramedic

## Abstract

**Background:**

Search filters aid clinicians and academics to accurately locate literature. Despite this, there is no search filter or Medical Subject Headings (MeSH) term pertaining to paramedics. Therefore, the aim of this study was to create two filters to meet to different needs of paramedic clinicians and academics.

**Methods:**

We created a gold standard from a reference set, which we measured against single terms and search filters. The words and phrases used stemmed from selective exclusion of terms from the previously published Prehospital Search Filter 2.0 as well as a Delphi session with an expert panel of paramedic researchers. Independent authors deemed articles paramedic-relevant or not following an agreed definition. We measured sensitivity, specificity, accuracy and number needed to read (NNR).

**Results:**

We located 2102 articles of which 431 (20.5%) related to paramedics. The performance of single terms was on average of high specificity (97.1% (Standard Deviation 7.4%), but of poor sensitivity (12.0%, SD 18.7%). The NNR ranged from 1 to 8.6. The sensitivity-maximising search filter yielded 98.4% sensitivity, with a specificity of 74.3% and a NNR of 2. The specificity-maximising filter achieved 88.3% in specificity, which only lowered the sensitivity to 94.7%, and thus a NNR of 1.48.

**Conclusions:**

We have created the first two paramedic specific search filters, one optimised for sensitivity and one optimised for specificity. The sensitivity-maximising search filter yielded 98.4% sensitivity, and a NNR of 2. The specificity-maximising filter achieved 88.3% in specificity, which only lowered the sensitivity to 94.7%, and a NNR of 1.48. A paramedic MeSH term is needed.

**Electronic supplementary material:**

The online version of this article (10.1186/s12911-017-0544-z) contains supplementary material, which is available to authorized users.

## Background

Over the past decade paramedic scope of practice and clinical responsibility have expanded significantly across most jurisdictions around the world [1]. Advanced clinical interventions traditionally performed exclusively by physicians, such as endotracheal intubation, ultrasound and thoracostomy, are increasingly becoming a part of the paramedic skill set [1]. Furthermore the role of extended care paramedics is increasingly taken up across Australia, New Zealand and the United Kingdom [2, 3]. The introduction of ‘*treat and refer’* guidelines are facilitating assessment and management provided exclusively by paramedics without eventual conveyance to an emergency department (ED) [4–6]. As such, paramedic specific research is more important than ever, necessitating a search filter.

The evolution of paramedics from vocationally-trained ‘ambulance drivers’ scripted by rigid protocols, to university-educated out-of-hospital clinicians has prompted recognition of the importance of evidence-based practice (EBP) and research into paramedic clinical practice [7]. The ever changing nature of paramedics in terms of clinical practice demands ongoing need for identification and appraisal of the literature [8]. As paramedics in a number of countries embark on campaigns for professional recognition via professional registration and or national examinations, the centrality of an EBP framework in paramedic practice is more important than ever [9].

Efficient retrieval of research is fundamental to EBP and is a prerequisite for the translation of evidence into practice and education [10–12]. Paramedic students, clinicians and researchers must be able to efficiently navigate the large volumes of published literature to find information relevant to their field. A limited understanding of formulating effective search strategies and time constraints can present a significant barrier to conducting research [13]. A strategy to overcome such challenges is the use of an adequate search filter. A search filter pertaining to paramedics specifically would serve to address one of the core concepts when creating an answerable question as part of the research process.

Search filters were first introduced as a tool in electronic database searching three decades ago [14]. Filters are a strategically developed string of search terms that restrict all the potentially retrieved articles to a particular subject/field (e.g. Palliative care) or methodology (e.g. Randomised Control Trials) [14]. The search filter is then combined with a research or clinical query to yield a set of relevant search results. It is important that it is sensitive enough to pick up all the relevant studies, but at the same time specific enough to avoid unnecessary retrieval of irrelevant literature [15]. How these two objectives are balanced is dependent on the intended use of the search filter. Search filters often offer an improved sensitivity and reduced number needed to read (NNR) [16], that is to say search filters can capture more relevant articles, whilst at the same time reducing the number of articles that has to be screened for secondary eligibility. The NNR is analogous to the number needed to treat (NNT) in clinical practice and intuitively describes how many articles have to be read in order to find one relating to paramedics [17].

Subject search filters have been developed for a variety of fields [14, 16, 18] and through various methodologies and “generations” [19]. There are three “generations” of search filter development. These generations are not purely distinct from each other and there is no natural progression from first to third generation filters. Choosing the filter development strategy therefore relies on the intended use of the filter and the current state of the field. First generation filters are created subjectively and not measured in terms of its’ performance. Second generation filters are like first generation in terms of their synthesis, however performance calculations are included. Third generation filters consist of a more objective approach through the use of methods like word frequency analysis and logistic regression. For instance, Gill et al. [15] developed two search filters (sensitivity-maximising and specificity-maximising) relating to primary care, whereby a set of gold standard articles underwent textual analysis for frequently occurring words and phrases (i.e. 3rd generation filter development) [19].

An existing search filter relating to paramedicine is the pre-hospital filter [8, 20]. The original pre-hospital filter, developed in 2004 for The Cochrane Library, was aimed at generating a comprehensive and transparent method for locating potentially relevant studies. The authors aimed to identify any research conducted in the pre-hospital setting. The authors of the original filter expressed the need for periodic updating of the filter in parallel with the evolving literature and scope of pre-hospital care. Such an update was conducted in 2010 by Burgess et al. [8] By not developing a gold standard set of relevant records to compare the filter performance against makes this a first generation filter development [19].

The existing pre-hospital filter however may have limited use for paramedics as it does not define *who* the research pertains to (e.g. paramedics), but rather *where* in relation to the hospital the care is delivered (e.g. pre-hospital versus in-hospital). For example, there are doctors and other healthcare personnel working in the out-of-hospital environment (e.g. physicians in Europe), and, though less often, paramedics working in hospitals. Secondly, the pre-hospital filter includes several terms relating to dispatch, public access defibrillation, and military medicine, which may not be specifically relevant to paramedics but are included deliberately to keep the filter broad. The pre-hospital filter may therefore potentially not be optimally representative of the paramedic literature.

Therefore we set out to create and determine the effectiveness of two paramedic literature search filters, through second generation filter development [19]. We aimed to create one filter that is sensitivity-maximising (i.e. broad and optimised for researchers [15]) and one specificity-maximising (i.e. narrow and optimised for clinicians [15]).

## Methods

We used the *Search Filter Appraisal Checklist* published by the UK Intertask Information Specialist subgroup to guide our method design and identify study limitations [21]. We relied on previously described, well-established methods for developing search filters [14–16]. We created a *gold standard* of labelled articles from a reference set, which we could then compare the performance of single terms and multiple terms (i.e. search filters) against. Performance was measured in relation to the filters effectiveness at retrieving relevant records and expressed in terms of sensitivity, specificity, and the NNR.

### Gold standard development

#### Retrieval set

Formulation of the ‘gold standard’ occurred via hand searching of a strategically selected pool of journal articles indexed in the MEDLINE database. The retrieval set included all articles published by six different journals (*Emergency Medicine Australasia*, *Resuscitation*, *Prehospital Emergency Care*, *Prehospital & Disaster Medicine*, *Air Medical Journal* and *European Journal of Emergency Medicine)* that were indexed during four different years (2006, 2009, 2012 & 2015). These journals and time periods were selected in an attempt to achieve a sample of articles that best represented the wider paramedic literature. We endeavoured to capture regional variances in the description of paramedics as well as the changes in vocabulary that have occurred over time such as ‘ambulance driver’ to ‘extended care paramedic’. Duplicates and articles without an abstract were excluded in preparation for screening.

#### Defining ‘paramedic’

Defining the term paramedic and deciding what literature pertains to a paramedic was a key challenge in the development of this filter. For the purpose of the filter development we defined a paramedic broadly, to include any out-of-hospital non-physician healthcare provider with any educational level or experience. Articles determined to be explicitly by, for, or about paramedics, were marked as paramedic relevant papers. This included papers that concerned a population group that included paramedics (e.g. physicians and paramedics, nurses and paramedics).

Pertinent exclusion criteria included articles on paramedic-related topics such as cardiopulmonary resuscitation, out-of-hospital cardiac arrest, triage, medical dispatch and disaster medicine that did not clearly address these subjects in a paramedic context. In deciding on uncertain articles, we kept in mind the final product, a paramedic search filter, which researchers and clinicians combine with their own search concepts.

#### Screening

Using the agreed upon criteria and mutual understanding of what defined a paramedic, the title and abstract of each article in the retrieval set was independently assessed by two authors (WS, AOO) to determine whether or not it related to paramedics. Inconsistencies were appraised by a third author (AO) reading the full text, consulting with the original reviewer if required and then making a final decision. Regular meetings were conducted during the early stages of screening to clarify uncertainties about inclusion and exclusion criteria. Endnote X7 was used to manage citations and record screening decisions, including the identification of inconsistencies.

### Filter development

#### Term selection

In order to identify key words and phrases used to search for paramedic specific literature, we referred to the previously published Prehospital Search Filter 2.0 [8]. By removing terms that exclusively related to the location of the clinician (e.g. out-of-hospital), we were left with search terms potentially relating to paramedics. Further identification of terms was achieved by conducting a Delphi session with an expert panel of seven paramedic academics and clinicians. The clinicians selected are full time paramedics working within our state (Victoria), while the selected academics comprised of experts internationally from our network from Australia, United States of America and the United Kingdom.

#### Single term analysis

The gold standard was exported from EndNote to Ovid MEDLINE using *unique identifier* numbers. A two-by-two contingency table was constructed (Table 1) to receive input of results from each filter being tested. In order for statistical descriptors to be calculated, two figures needed to be found; a + b and a. a + b is the number of articles that were common to the experimental filter results and the screened reference set. These articles could have been paramedic or non-paramedic articles but must have been articles from the reference set. In simple terms, a + b is the subset of the reference set that was returned by the experimental filter. a is the number of articles that were common to experimental filter results and the gold standard. These numbers were found using the Boolean operator AND in MEDLINE. The remainder of the contingency table could then be completed by inference and statistical analysis recorded.

**Table 1:** Contingency table comparing filter to ‘reference set’ and explanation of statistical descriptions

Filter	Manual review of each article		
	Paramedic articles	Non-paramedic articles	
Article identified	a	b	a + b
Article not identified	c	d	c + d
	a + c	b + d	a + b + c + d

Caption: Sensitivity = a/(a + c): proportion of all articles relevant to paramedics in the reference set that are retrieved by the filter. Specificity = d/(b + d): proportion of all articles not relevant to paramedics in the reference set that are correctly not retrieved by the filter. Precision = a/(a + b). NNR = 1/precision: the number of relevant and non-relevant articles that need to be screened in order to find one of relevance

#### Combined term filters

The *combined term filters* were developed by manually testing iterations of term combinations. High performing words from single term analysis were combined in MEDLINE using the Boolean operator OR. Terms were added and subtracted from trial filters until optimal performance was achieved. This process was repeated twice. Once to achieve a filter that favoured sensitivity (sensitivity-maximising) and once to achieve a filter that favoured specificity (specificity-maximising). The optimal search has both high sensitivity and high precision whilst keeping the NNR low [16].

#### Statistical descriptions

We expressed the performance of the filters in terms of sensitivity, specificity and NNR.

## Results

**Fig. 1:**
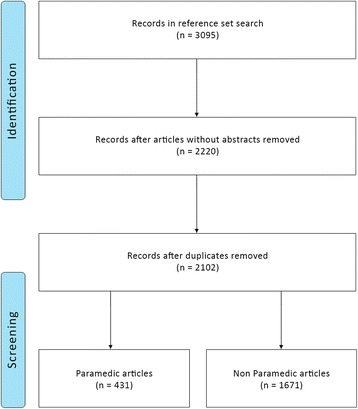
Screening process and result

In creating the gold standard the initial search yielded 3095 articles of which 2102 were left after duplicates and articles without abstracts were removed. Those 2102 articles were screened, of which 431 (20.5%) related to paramedics. (Fig. 1).

The sensitivity, specificity and NNR for each of the single terms are presented in Additional file 1. Unsurprisingly, single terms perform generally with high specificity, but poor sensitivity and subsequent high NNR. The mean sensitivity for the single terms was 12.0% (SD 18.7), whilst the mean specificity was 97.1% (SD 7.4%) with the NNR ranging from 1 to 8.6.

Regarding the combined term analysis (i.e. search filters) high sensitivity was achieved, with acceptable specificity. The sensitivity-maximising search filter yielded 98.4% sensitivity, but with a specificity of 74.3%, the NNR was 2. The specificity-maximising filter achieved 88.3% in specificity, which only lowered the sensitivity to 94.7%, and thus a NNR of 1.48. (Table 2) The pre-hospital filter had lower sensitivity than the sensitivity-maximising paramedic search filter and the highest NNR of all filters.

**Table 2:** Performance of the two paramedic search filters as well as the existing prehospital filter

Filter	Performance		
	Sensitivity	Specificity	NNR
Paramedic Filter (optimised for **sensitivity**) *Ambulances.sh OR Emergency Medical Technicians.sh OR Air Ambulances.sh OR* ***emergency medical services.sh*** OR paramedic*.tw OR ems.tw OR emt.tw OR prehospital.tw OR pre-hospital.tw OR first responder*.tw OR emergency medical technicians.tw OR emergency services.tw OR Ambulance*.tw OR HEMS.tw OR field triage.tw OR ***out-of-hospital.tw***	98.4%	74.3%	2.00
Paramedic Filter (optimised for **specificity**) Ambulances.sh OR Emergency Medical Technicians.sh OR Air Ambulances.sh OR paramedic*.tw OR ems.tw OR emt.tw OR prehospital.tw OR pre-hospital.tw OR first responder*.tw OR emergency medical technicians.tw OR emergency services.tw OR Ambulance*.tw OR HEMS.tw OR field triage.tw	94.7%	88.3%	1.48
Prehospital filter [8]	97.4%	65.4%	2.44

Additional terms that are unique to the sensitivity optimised filter are bolded

## Discussion

The two filters herein are the first search filters relating to paramedics. In accordance with filter developments in other fields, we have provided i) a sensitive filter, which is optimal for use by researchers with a necessary high NNR in order to capture all relevant articles, and ii) a specific filter, which is optimal for clinicians, students, and others who accept a search strategy which may not identify all relevant papers, but at the benefit of reducing the NNR. The search filters are naturally not intended for use in the field at the point of care, but rather to advance the academic paramedicine profession and more accurately guide clinicians with an interest in the literature.

Search filters and diagnostic tests are analogous [15]. They both deal with the struggle of choosing between false negatives and false positives. A search filter or a diagnostic test with high specificity chiefly identifies relevant papers or patients (i.e. low number of false positives). On the other hand, a search filter or a diagnostic test with high sensitivity identifies almost every relevant result, but does so at the expense of including a lot of irrelevant results (i.e. high number of false positives). Therefore, when a highly specific filter is positive it ‘rules in’, whilst a highly sensitive filter, when negative, ‘rules it out’. The ideal test, diagnostic or search filter, maximises both specificity and sensitivity. However, any filter may be criticised for being too specific or not sensitive enough, and a balance is therefore sought after [14]. The determination of where the balance should lie on depends on the intended use of the filter. A solution to overcome the undesirable compromise, as we have offered in this paper, is to generate two different filters to suit two different user groups.

Currently there is no MeSH term pertaining to paramedics. As such, search filters are necessary to facilitate research. In order to recognise paramedicine as a profession and continue the push towards paramedic-specific EBP, a paramedic MeSH term is both warranted and timely. This term should ideally include anything from EMTs to advanced care out-of-hospital paramedics. As noted by Sladek et al. [14] MeSH terms yield high specificity, but low or extremely low sensitivity. For MeSH terms pertaining to palliative care, sensitivity ranged from 29.1 to 0.7%, whilst consistently keeping specificity scores above 99%. The poor sensitivity of MeSH terms partly stems from how a field is conceptualised and described, [14] as well as when the field has a broad definitions (e.g. primary care) [15]. It is therefore anticipated that a paramedic MeSH term would have very low sensitivity as it is both described vastly differently and broadly across the globe.

Some issues that explain the difficulty in finding the right balance is the term ‘paramedic’. Paramedic as a term is used vastly different around the world, and at different time points. For instance, in the USA, EMS and EMTs are the main out-of-hospital operators, with some having Bachelor degrees, whereas in Australia Bachelor degrees are required by all paramedics, with some completing additional higher degree qualifications including Masters and PhD. The airwing or helicopter service as part of an emergency service are sometimes staffed by physicians and assistants (e.g. in Europe) or by paramedics (e.g. Australia). Furthermore, the rapidly changing scope of practice (e.g. from first aid to advanced out-of-hospital resuscitation) and terminology (e.g. from ambulance driver to extended care paramedic) of paramedics over time may be difficult to capture in a single search filter. The filters will therefore need updating as new scopes of practice and terms describing ‘paramedics’ come to light.

It is clear that clinicians, despite best intentions, possibly will not spend large amounts of time on trawling through database searching [22]. Searching the primary literature remains important, even in today’s prevalence of evidence synthesis tools [15], however the task is becoming more difficult given the inevitable increasing amount of literature available [23]. A paramedic filter which offers a low NNR would serve to provide the busy clinician with mostly relevant articles.

It must be recognised that this convenience comes with a drawback, and that is the uncertainty of not obtaining all the relevant articles. Whilst a search filter that is specific may be used in everyday clinical practice, it is not sufficient for use when answering academic questions, which requires a systematic, comprehensive and balanced answer.

One of the challenges for this project was defining a paramedic. What literature pertains to paramedics, and who is a paramedic, Emergency Medical Technician and Mobile Intensive care? What about military medicine? Our search filter is pertaining to a specific group of clinicians worldwide and as such is challenging to clearly dichotomise. This is in contrast to methodological search filters, in which there is minimal confusion as to whether the study is of a certain quality (e.g. Randomised Control Trial) or pertains to certain patient cohorts (e.g. paediatrics). Future direction should include development of a paramedic MeSH term and ‘third-generation’ filter development whereby the filter is validated in a separate pool of articles from which it was developed (external validation), as well as more systematically refined (e.g. through bootstrapping methods).

## Conclusion

We have created two paramedic specific search filters, one for optimised sensitivity and one for optimised specificity. As such, the filters suggested in this paper offer the options of either i) capturing the majority of paramedic relevant articles or ii) reading mainly paramedic relevant articles (i.e. a low NNR). A paramedic MeSH term is needed, but firstly requires a universal definition of what a paramedic constitutes.

## Additional file

Additional file 1: Overview of the single term analysis. Table providing overview of the single term analysis. (DOCX 83 kb)

## Abbreviations

EBP: Evidence based practice
ED: Emergency Department
MeSH: Medical subject headings
NNR: Number needed to read
NNT: Number needed to treat
SD: Standard deviation
