# Supplementary material for: Paramedic literature search filters: optimised for clinicians and academics
Source: BMC Med Inform Decis Mak. 2017 Oct 11;17:146. doi: 10.1186/s12911-017-0544-z (PMC5637081; doi:10.1186/s12911-017-0544-z)
Supplement: Additional file 1: — Overview of the single term analysis. Table providing overview of the single term analysis. (DOCX 83 kb) [file 12911_2017_544_MOESM1_ESM.docx]

# Additional file 1: Single Term Analysis Results

| **Filter** | **Performance** | | |
| --- | --- | --- | --- |
|  | **Sensitivity** | **Specificity** | **NNR** |
| Aviation Medic.tw | - | - | - |
| Adult Retrieval.tw | - | - | - |
| Ambulance*.tw | 25.3% | 97.6% | 1.37 |
| Ambulances.sh | 11.6% | 99.4% | 1.20 |
| Ambulance Technician.tw | - | - | - |
| Ambulance Volunteer.tw | - | - | - |
| Advanced EMS provider.tw | - | - | - |
| Advanced EMS personnel.tw | - | - | - |
| Air Ambulances.sh | 8.35% | 98.6% | 1.64 |
| Army Medic.tw | - | - | - |
| Aviation Medic.tw | - | - | - |
| Clinical Transport.tw | - | - | - |
| Clinical Support Officer.tw | - | - | - |
| CSO.tw | - | - | - |
| EMS.tw | 48.3% | 97.8% | 1.17 |
| EMT.tw | 4.40% | 100% | 1.00 |
| EMS provider.tw | 2.32% | 100% | 1.00 |
| EMS personnel.tw | 6.26% | 99.8% | 1.15 |
| Emergency.tw | 67.9% | 58.6% | 3.36 |
| Emergency Medical Services.tw | 40.1% | 98.1% | 1.18 |
| Emergency Medical Services.sh | 65.4% | 87.6% | 1.73 |
| Emergency Medical Technicians.tw | 6.26% | 99.9% | 1.04 |
| Emergency Medical Technicians.sh | 19.3% | 99.4% | 1.11 |
| Emergency Medicine.sh | 3.02% | 94.1% | 8.62 |
| Emergency practitioner.tw | - | - | - |
| Emergency Services.tw | 1.39% | 99.5% | 2.33 |
| Emergency Technician*.tw | 0.23% | 100% | 1.00 |
| Emergency Treatment.sh | 2.09% | 97.8% | 5.00 |
| Extended Field Care.tw | - | - | - |
| First Aid.tw | 2.09% | 99.3% | 2.33 |
| First Aid.sh | 0.70% | 99.5% | 3.67 |
| First responder*.tw | 3.71% | 98.7% | 2.31 |
| Fire rescue.tw | 0.00% | 99.9% | # |
| field care.tw | 0.00% | 99.9% | # |
| field medicine.tw | - | - | - |
| Field triage.tw | 2.32% | 99.82% | 1.30 |
| HEMS.tw | 4.18% | 99.8% | 1.22 |
| Helicopter Emergency Medical Service.tw | 1.62% | 99.9% | 1.29 |
| Inter-hospital transfer.tw | 0.00% | 99.8% | - |
| Medical direct*.tw | 3.02% | 99.9% | 1.15 |
| Medic.tw | 0.47% | 100% | 1.00 |
| Mine First Aid Officer.tw | - | - | - |
| Mine Rescue Service.tw | - | - | - |
| Military Medicine.sh | 0.23% | 99.9% | 3.00 |
| Neonatal retrieval.tw | - | - | - |
| Navy Medic.tw | - | - | - |
| OHCA.tw | 8.35% | 95.2% | 3.22 |
| Out-of-hospital.tw | 23.0% | 88.6% | 2.92 |
| Pre-hospital.tw | 4.87% | 98.7% | 2.00 |
| Prehospital.tw | 39.7% | 95.7% | 1.42 |
| Paramedicine.tw | 0.23% | 100% | 1.00 |
| Prolonged Field Care.tw | - | - | - |
| Patrol medic.tw | - | - | - |
| Patient retrieval.tw | - | - | - |
| Patient Transport Service.tw | - | - | - |
| Paediatric retrieval.tw | - | - | - |
| PTS.tw | 0.00% | 99.9% | # |
| Volunteer Ambulance Officer.tw | - | - | - |
| VAO.tw | - | - | - |

# - NNR could not be defined when precision was equal to 0.00%
